# Mechanical Properties and Strength Reliability of Impregnated Wood after High Temperature Conditions

**DOI:** 10.3390/ma13235521

**Published:** 2020-12-03

**Authors:** Krzysztof Przystupa, Daniel Pieniak, Waldemar Samociuk, Agata Walczak, Grzegorz Bartnik, Renata Kamocka-Bronisz, Monika Sutuła

**Affiliations:** 1 Department of Automation, Lublin University of Technology, Nadbystrzycka 36, 20-618 Lublin, Poland; 2 The Main School of Fire Service, Faculty of Safety Engineering and Civil Protection, Slowackiego 52/54, 01-629 Warsaw, Poland; daniel.pieniak@wsei.lublin.pl (D.P.); awalczak@sgsp.edu.pl (A.W.); rkamocka@sgsp.edu.pl (R.K.-B.); msutula@sgsp.edu.pl (M.S.); 3 Department of Mechanical Engineering and Automatics, University of Life Sciences in Lublin, 20-612 Lublin, Poland; waldemar.samociuk@up.lublin.pl; 4 Faculty of Transport and Computer Science, University of Economics and Innovations in Lublin, Projektowa 4, 20-209 Lublin, Poland; grzegorz.bartnik@wsei.lublin.pl

**Keywords:** wood impregnation, bending strength, strength reliability

## Abstract

The paper presents the results of the research into the impact of impregnation of wood on its bending strength and elastic modulus under normal conditions and after thermal treatment and investigates its structural reliability. Pinewood, non-impregnated and pressure impregnated with a solution with SiO_2_ nanoparticles, was used in this research. The use of nanoparticles decreases the flammability of timber among others. Some of the tested samples were treated at 250 °C. This temperature corresponds to the boundary of the self-ignition of wood. This elevated temperature was assumed to be reached by a given speed of heating within 10 min, and then the samples were stored in these conditions for 10 and 20 min. The tests demonstrate that the bending strength of the impregnated wood was slightly improved, the impregnation did not impact the elastic modulus of the material in all such conditions, and the residual strength decreased less for the wood impregnated after being exposed to the elevated temperatures. The reliability analysis proves a positive effect of impregnation with a solution with SiO_2_ on the durability of wood, both after being exposed to normal and elevated temperatures. The distribution of the failure rates indicates a more intensive degradation of non-impregnated wood. The distribution of the survival function demonstrates a more probable non-destruction of impregnated wood after elevated temperature conditions.

## 1. Introduction

Since ancient times, wood has been one of the most popular building materials and materials for artistic works [1]. Wood is a natural composite material composed mainly of cellulose, hemicellulose and lignin [2] and shows many advantages as a building material. Timber shows beneficial physical and technological properties, has a relatively high strength-to-weight ratio and low density. Wood is a renewable and ecological material, easily processed, available in many sizes, shapes and colors [2,3]. It has low volumetric weight, poor thermal and electrical conductivity and good sonic conductivity [3]. Wood, above all, shows good physical and mechanical properties [4]. It should be stressed, however, that strength of wood depends on many factors, including types of wood, the direction of forces acting on fibers, humidity and specific weight, anatomical structure and defects of wood [5].

The durability of wood depends on several factors such as humidity, the presence of fungi and insects, and many more. To avoid its degradation, wood should be repaired, maintained or replaced before its lifetime ends [6]. One of the available methods is constructive wood protection. If it is only painted or varnished and not impregnated, its surface is only protected against photochemical degradation, dimensional changes, biological factors and fire for a maximum of about 2 years [7] so chemical agents to improve physical, mechanical, biological and flame retardant properties of wood have been recently increasingly and more willingly investigated [4,8]. Wood contains cellulose composed of carbon so it burns easily in fire or if exposed to a heat stream [4]. Thermal degradation of dried cellulose occurs at a temperature of about 300 °C, but the degradation of hemicellulose begins as early as in the temperatures ranging from 150 to 200 °C. Lignin that makes a wood structure cohesive decomposes in temperatures from 220 °C to 250 °C and dehydrates at 200 °C. The mechanism of burning begins at a temperature of about 105 °C just when free water evaporates from wood. Above 200 °C, intensive gas emissions occur because exothermic reactions begin and wood is intensely discolored to become dark brown. This process accelerates if the temperature of ~250 °C is exceeded [9]. When wood burns, no harmful substances are released, but a charred layer is formed and becomes thicker and thicker as fire lasts. The charred layer is a kind of barrier for the front of heat penetrating into the structure of the construction element so wood burns more slowly until this charred layer is damaged or cracked [10,11]. The formation of such a charred layer leads to a reduced effective cross-section by this charred part [12]. A section in a timber core is responsible for timber residual load-bearing capacity. Fire, however, may not be neutral to a timber core section. The temperature of timber in a non-charred section may reach 120–250 °C, depending on a type and power of fire and the degree of its progress [13]. Fireproof impregnants are the most common impregnants to protect wood from high temperatures. Such impregnants should prevent a loss of bearing capacity by accelerating the formation of a charred layer that protects a timber core section and limits spreading flames. Impregnants are classified into two groups reflecting methods of their application [14]. The former group is impregnants penetrating into wood which most often have salt agents. Such fireproof impregnants are concentrated aqueous solutions used for a deep saturation of a wooden element using a vacuum or vacuum-pressure method. This group includes agents composed of compounds of phosphorus, boron, magnesium, ammonium, nitrogen and urea. The latter group represents surface-acting impregnants as paints, varnishes, water solutions, and thin plates. These agents form a protective layer on a wooden surface [15]. Despite the growing interest in chemical wood preservatives, their impact on mechanical properties is hardly described. The previous investigations show that salt impregnants improve compressive strength from 4.6 to 9.6% and decrease bending strength from 2.9% to 16% [16]. Bendtsen’s research shows that ammoniacal copper arsenate (ACA) and copper-chromium arsenate do not significantly change an elastic modulus [17]. Generally, the impact of an impregnant on mechanical properties depends on type of impregnant, impregnated material, impregnation methods and time.

Structural reliability under fire conditions depends, e.g., on how long a non-charred core of construction elements is capable of preserving their load-bearing capacity and stiffness. Improved reliability may mean more time for evacuation, rescue and firefighting, including making a temporary stabilization of construction elements [18]. The construction industry uses the concept of structural reliability. Structural reliability is defined as the ability of a structure to function without failure during its expected life; the contractual probability of structure survival. The postulated structural reliability is achieved by satisfying design criteria and technical requirements for a given structure, specified in relevant normative documents [19]. According to this definition, the construction material must conform to predetermined criteria. Incompatibility with these criteria means that such materials are unreliable. The notion of incompatibility is multi-categorial and applies to categories covering different types of biological, strength, durability, functional, aesthetic and other incompatibilities. As one of the categories of inability, incompatibility may occur not only when an object is used but also in all other phases of its life cycle [20]. Structural reliability can be considered in terms of the impact of individual elements of a structure and their properties which are fundamental for the user [21,22,23,24]. There are three levels of analysis: the level of points—more specifically particles of structural material, the level of sections—i.e., a cross-section of a structural element, and the level of objects, or a structural system (construction). [25,26]. Reliability for the mechanical strength of materials was discussed in papers [27,28]. M. Warszyński [29] defines the reliability of an object or element as its ability to carry loads under specified conditions and over specified time periods while maintaining its required strength. Generally, structures are protected by stabilizing structural elements.

Bearing in mind the above, this study evaluates the impact of vacuum fire retardant impregnation on strength and elastic modulus. These tests refer to the general purpose of the work, which is the assessment of strength reliability of solid pine wood after high-temperature conditions.

## 2. Material and Research Method

Samples for strength testing were Scots pine (Latin: Pinus sylvestris). It was homogeneous wood without knots and other defects. All of the samples were machined using the same mechanical method under identical processing conditions. Ninety samples were used in the research. Fifteen samples were tested in each group. Half of the samples were impregnated with a solution of 400 mg/L water and SiO_2_ nanoparticles (Sigma—Aldrich, Darmstadt, Germany) (Table 1). The solution was poured into a deep container to completely cover the samples for 20 min. The samples were protected against flames with a method of pressure-impregnation. They were vacuum impregnated in the SPU-200 vacuum dryer of an operating temperature range from ambient temperature to 200 °C and a permissible vacuum of 0.099 MPa. The samples were kept in the chamber for 15 min at a vacuum of 0.6 atm. Such prepared samples were then dried to ambient temperature.

Half of the impregnated and non-impregnated samples were high temperature treated. The temperature range of the experiment and a minimum time period of exposure of the samples to the elevated temperatures were determined in the preliminary tests. This time period is necessary to obtain an even temperature in the volume of the sample. The temperature inside the material was specified with a thermocouple placed in a hole drilled in the samples. This was the method for measuring the temperature in the geometric center of the sample. The minimum heating time of the sample was specified as the time after which the thermocouple placed inside the sample allowed for measuring the temperature specified in the research plan. An ambient temperature of 20 °C was adopted as a starting point for the preliminary tests. A boundary temperature was set as 250 °C.

**Figure 1:**
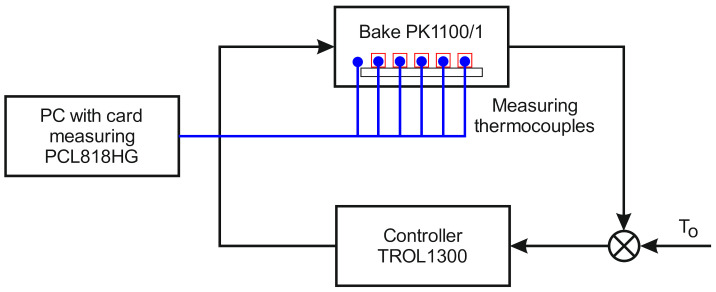
The scheme of the test stand for heating.

The basic instrument of the test stand for heating was a PK 1100/5 average-temperature chamber furnace (Figure 1). The samples were placed inside the furnace chamber and the measurement thermocouples were mounted on the external surfaces of two selected samples. The temperature around the samples inside the furnace was also measured during the tests based on a standard temperature–time curve.

**Figure 2:**
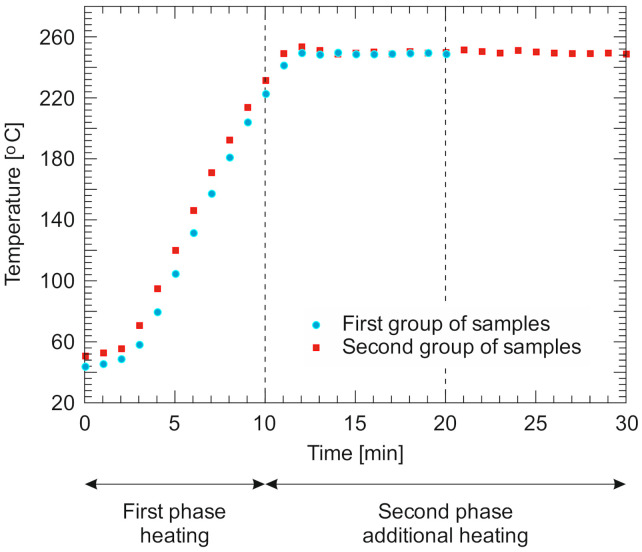
Temperature–time measured with the thermocouples mounted on the surfaces of the samples.

The heating of the samples proceeded in two phases. The samples were heated up to 250 °C in the first 10-min phase. After this time, the second phase followed. Half of the samples were annealed at the set temperature for 10 min. This time was the minimum time to obtain a temperature of 250 °C in the entire volume of the sample. The other half of the samples was heated in the second phase at 250 °C for 20 min, which is significantly longer than for the temperature equilibrium over the entire volume of the first group of samples. The high temperature treatments of both groups of the samples are depicted in the temperature–time diagram (Figure 2).

The heating, in both cases, was followed by taking the samples out of the furnace and cooling them naturally outside the chamber to achieve the ambient temperature of the laboratory of approx. 20 °C.

**Figure 3:**
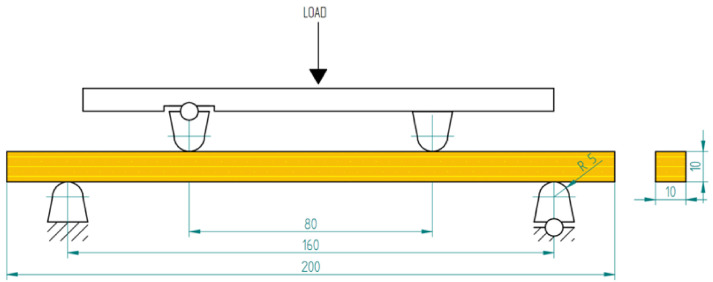
Four-point bending strength test of wood.

The bending strength test was carried out with a Zwick/Roell Z100 universal testing machine (Ulm, Germany) and a head of a nominal force of 10 kN. A four-point bending test across the fibres was carried out for the samples of 200 mm × 10 mm × 10 mm (Figure 3). The dimension of the sample was selected so that the entire volume of the sample could be impregnated. The cross-sectional dimensions of the samples in all test groups were measured. The test was done under quasi-static loads at a speed of 1 mm/min and for an elastic modulus specified in the range from 10 to 40% of the maximum force.

The time of the reliability analysis was measured from the moment of the first fracture in the beam until the moment of its destruction. This time is important in rescue, firefighting and protective actions in the post-fire period [11]. The appearance of the fracture was manifested by a stepping force drop in the stress–strain curve and a characteristic acoustic effect. The reliability analysis was multi-stage and Weibull grids were used. The parameters of 2-parameter Weibull distribution were estimated with the maximum likelihood method [30,31]. The parameters of shape and scale of distribution were determined from the grids. The parameter of scale is the time period until 63.2% of the samples will be destructed and the parameter of shape determines the preservation of probability of destruction over time.

The Kaplan–Meier estimator (survival function) is the ratio of the number of observed objects that remained in the state of indestructibility in the time t to the initial number of the objects (samples). It is a cumulative proportion of cases (CPS—Cumulative Probability of Surviving) that have not reached the boundary state from the time of the first clear cracks until the time under consideration [32]. In Equation (1), *S(t)* is the estimated survival function, *n* is the total number of cases and Π is the product of all cases less than or equal to *t*; *δ(j)* is a constant equal to 1 [32].

(1) $$S\left(t\right)=\prod {\left(\frac{n-j}{n-j+1}\right)}^{\delta \left(j\right)}$$

The failure rate, i.e., damage intensity [33] refers to the Weibull equation. The function *h(t)* for the Weibull distribution is calculated from Equation (2) (with the positive parameters *b*, *c*, and θ) [34]:

(2) $$h\left(t\right)=\frac{f\left(t\right)}{R\left(t\right)}=\frac{\left(c{\left(t-\theta \right)}^{c-1}\right)}{b^{c}}$$

where:

*t*—generalized time,
*b*—parameter of scale,
*c*—parameter of shape,
θ—parameter of location, (0 for the 2-parameter Weibull distribution, which is explained in the discussion on the research results).

The values of the cumulative failure rate were determined from Equation (3):

(3) $$H\left(t\right)=\frac{\left(t-\theta \right)}{b^{c}}$$

where:

*t*—generalized time,
*b*—parameter of scale,
*c*—parameter of shape.

## 3. Results of Research and Discussion

### 3.1. Results of the Strength and Modulus of Elasticity in Tension Tests

Table 2 shows the results of the research into the modulus of elasticity (E—Young’s modulus) and the value of bending stresses at the moment of destruction (σ*_B_*) of non-impregnated (pine) and impregnated (pine i.) pine wood samples. The differences in the mean value, for each of the types of samples, indicate higher values of Young’s modulus and lower values of the coefficient of the variable for the impregnated samples. The bending stress tests for the non-impregnated samples, on the other hand, show that the differences in the tested values at different temperatures are higher than for the impregnated ones, and a dispersion of the results is also greater. As expected, the obtained results indicate a decrease in bending strength and Young’s modulus values in the samples heated at 250 °C compared to the ones tested at 20 °C. The strength values also decrease as the time of the high-temperature treatment increases, but these discrepancies are insignificant. During the tests, the humidity of the samples and their density were not measured. The samples were stored in the laboratory between measurements at a constant temperature of 20 °C. A_0_ [mm^2^] is the cross-section of samples, it is the recorded measure, identifying the geometrical properties of the samples before and after heat treatment. Abbreviated designations in Table 2 “s.dev” and “c.var” denote the standard deviation and the coefficient of variation, respectively.

### 3.2. Results of Structural Reliability Tests

Table 3 shows the values of parameters of shape and scale obtained from the Weibull grids. The parameters of 2-parameter Weibull distribution were estimated using the maximum likelihood method [30]. The maximum likelihood method assumes that the reliability of the *L* test of the *n* observation of *x1*, *x2*, *...*, *xn* is a function of the total probability *p(x1*, *x2*, *...*, *xn)* where *x1*, *x2*, *...*, *xn* are discrete random variables. If *x1*, *x2*, *...*, *xn* are continuous random variables, the reliability of the *L* test of the *n* observations of *x1*, *x2*, *...*, *xn* is the overall probability density function *f(x1*, *x2*, *...*, *xn)* [35]. The parameters of 2-parameter Weibull distribution were read from the graphs. The parameter of shape equals the coefficient of the slope of the matched straight line, and the parameter of scale can be calculated as *exp* (constant term/slope).

The results for the parameter of shape indicate small differences between the impregnated and non-impregnated wood for the samples tested both at 20 °C and 250 °C. The values of the parameter of scale show a clear difference between impregnated and non-impregnated wood in favor of impregnated one as the results indicate that the preserving of the strength parameters of the tested material is much more predictable.

**Figure 4:**
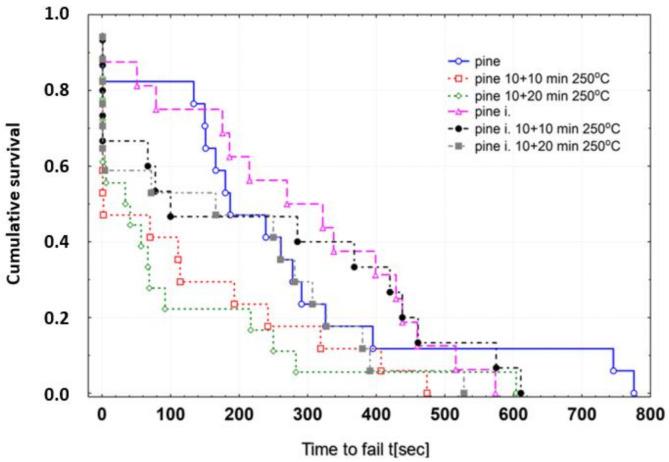
Distribution of the cumulative probability of survival as a function of time until destruction.

Figure 4 shows the distribution of the Kaplan–Meier survival curve. The courses of the correlations indicate more favorable values for the survival function of the impregnated wood for each type of test (at 20 °C and 250 °C for two time periods of heating), especially for the longer time period until destruction.

**Figure 5:**
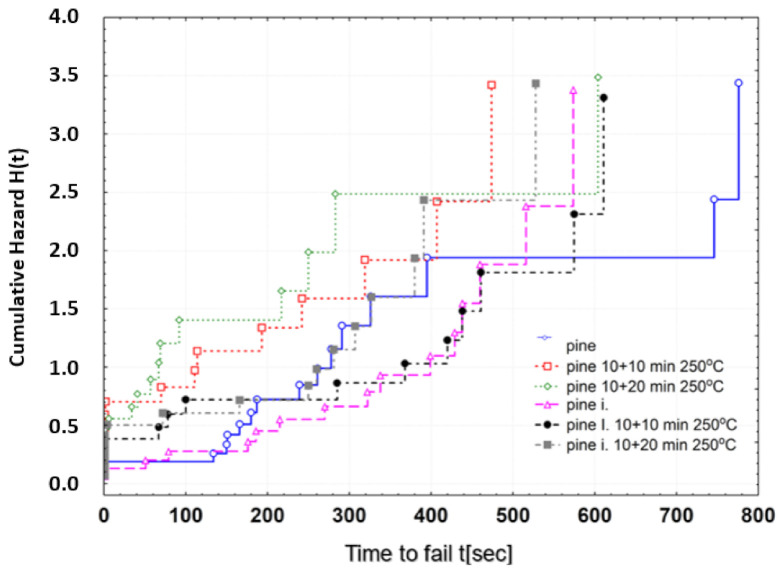
Distribution of risk (cumulative intensity of destruction probability) as a function of time until destruction.

Figure 5 shows the distribution of the failure rate. The courses of the correlations indicate that with the general increasing tendency of the failure rate as the time period until destruction increases, the courses are better for the impregnated wood, especially if heated at 250 °C when the risk of destruction is definitely higher for non-impregnated wood.

### 3.3. Discussion

Wooden structures are often treated as temporary structures to rebuild municipal infrastructure damaged by floods and other natural disasters [36]. Wood was also used instead of other, more durable and stronger building materials if not enough funds. Wood is also more common because of its numerous advantages and environmental reasons. Those factors contribute to the continuous improvement of properties of wood, i.e., its durability, strength and fire resistance [37,38,39]. This research issue, therefore, is undertaken for its practicality.

The study has demonstrated a slight impact of impregnation on the improvement of bending strength of wood and an impact of heating on strength of wood. A decrease in strength was recorded. It should be highlighted that the elongation of the high-temperature treatment time in the second phase from 10 to 20 min causes the decrease of the standard deviation of bending strength. This is opposite to the study by Soti et al. in which the strength value variation increases with the increase of time of exposure to elevated temperatures [40]. The residual strength of the impregnated wood after its exposure to the elevated temperature was higher. This effect is explained in specialist literature. Such a decrease in strength may result from a decrease in humidity due to thermal degradation of wood. This phenomenon proceeds more slowly in impregnated wood, causing the shortening of hydrogen bonds of polymer cellulose [41]. Cellulose represents the largest share of the volume of wood. This polymer is responsible for the mechanical strength of wood [42,43]. Hemicellulose, one of the cellulose derivatives, consists of branched amorphous polymers and fills the area between cellulose and lignin in the wood structure. Dried cellulose degrades at about 300 °C, whereas hemicellulose already at 150–200 °C [9,44]. Schaffer [45] claims that the strength of wood also depends on lignin that insulates wood fibers. Lignin is an amorphous polymer responsible for the cohesion of the wood structure.

The statistical Kruskal–Wallis test was performed due to the small differences in the average values of an elastic modulus in the groups of the non-impregnated and impregnated samples. This rank statistical test does not assume a normal distribution. It is sometimes considered as a non-parametric alternative to single-factor analysis of variance between groups [34]. This test showed no statistically significant differences in the elastic modulus in comparison to groups of the same parameters of heating (*p* > 0.05). The analysis shows that the impregnation method applied in our own research does not affect the elasticity of wood, and the impact of elevated temperatures is observed.

The level of reliability analysis is a critical issue in the reliability analysis of structures. Such an analysis can be conducted for deterministic static-strength evaluation and probabilistic evaluation of structural reliability. Three levels of this analysis include: the level of a point, more precisely a small volume of structural material, the level of a section, i.e., a section of a structural element, and the level of an object, i.e., a structural system [46] in reference to a time period from clear damage until destruction. The Weibull analysis was used to investigate the reliability of high-temperature treated wood corresponding to the state in the core section exposed to a thermal impact produced by fire. Weibull distribution parameters enable the flexible shaping of the distribution curve and estimation of the probability of destruction or non-destruction from a relatively small number of samples [47]. A 2-parameter Weibull distribution was used here. The parameter of location in a 3-parameter Weibull distribution can be defined as the highest non-destructive stress. This value is not known so the parameter of location is often assumed to be 0 and, consequently, a 2-parameter Weibull distribution is applied [48]. The same approach was followed in the reliability analysis discussed in this paper. The beneficial effect of SiO_2_ impregnation on destruction resistance was demonstrated. The impregnated beams were more reliable and higher parameters of shape were obtained both at the normal temperature and after high-temperature treatment. The values of the Weibull distribution of the parameter of scale obtained for the impregnated wood after the heating are particularly noteworthy. The values of this parameter are several times higher than those for the non-impregnated wood. Such a correlation is also indicated by the distribution of survival and failure rates. A higher intensity of destruction of the non-impregnated wood is shown.

**Figure 6:**
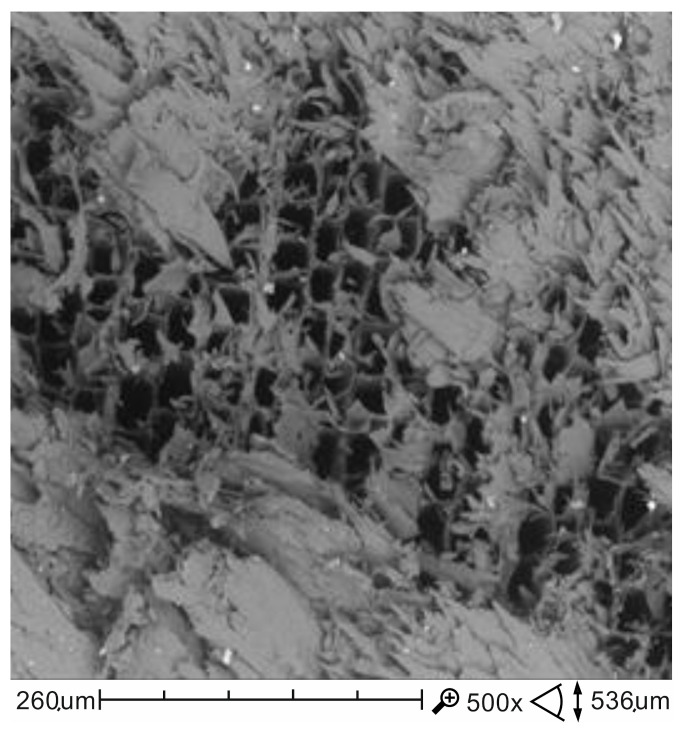
SEM images of the non-impregnated porous wood structure at a magnification of 500×.

**Figure 7:**
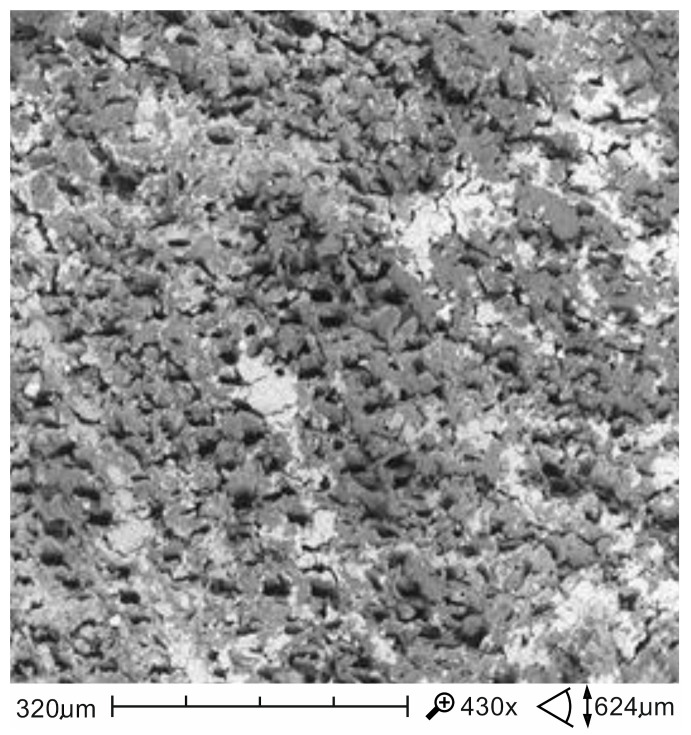
SEM images of the impregnated porous wood structure at a magnification of 430×.

The applied impregnant fulfils its functions while still maintaining a higher deformation capacity and a higher time of preservation of load-bearing capacity of wood over a destructive process. The results of the tests, positive for the impregnated samples, can be explained by observing the distribution of impregnation particles on the surfaces of wooden beams. The impregnant that fills the wood pores and the impregnation that also adhered to the cell walls of the wood are capable of insulating and sealing and, consequently, can slow down thermal degradation of structures deep into the wooden element and limit the emission of flammable gases (Figure 6 and Figure 7).

The discussed analysis is limited by a conclusion drawn only from the ad hoc strength properties of the samples without their loading history. Construction engineers generally know that the long-term strength of wood is much lower than the short-term. Another limitation is the size of samples corresponding to laboratory testing conditions. A wooden element loses its strength because of more defects as its size increase [49]. The study [50] demonstrates that the strength of materials increases as a characteristic dimension of microstructures to dimensions of structural elements made of this material increases, which depends on the size and distance of piled up local stress zones.

Despite these limitations, the results and analyses enabled us to achieve our research aims.

## 4. Conclusions

The following conclusions are drawn from the investigations and analyses:

1. The results of this type of research identify the effect of impregnation and an impregnation method on the performance of wood. The strength and reliability of wood impregnated with the nanoparticle agent is greater, probably due to impregnation from the upper layer of wood towards the core as well as its sealing. Degradation of silicon dioxide is long-term, but it is an inorganic compound commonly found in the Earth as a mineral, rock constituent and of a low environmental impact.
2. Thermal properties of silica are not insignificant in fire protection, e.g., its high melting point, low thermal conductivity.
3. The applied impregnant improves the strength reliability of solid wood tested in our research.

## Figures and Tables

**Table 1:** Detailed physical and chemical properties of SiO_2_ flame retardant impregnant.

Properties of the Impregnant	Description/Value
View	White powder
Scent	None
Particle size	10–20 nm
Initial melting point	1600 °C
Initial boiling point	2300 °C
Volumetric density	0.011 g/mL

**Table 2:** Statistics of the four-point bending strength test results.

Parameter	Material (N = 15)	Temp. [°C]	Exp. Time [min]	Mean [GPa]	S.dev. [MPa]	C.var. [%]
E [GPa]	Pine wood	20	-	9.04	0.99	11.05
		250	10 + 10	7.85	1.71	21.77
		250	10 + 20	7.51	1.51	20.10
	Pine wood, SiO_2_ impregnated	20	-	8.93	0.806	9.03
		250	10 + 10	7.67	1.48	19.29
		250	10 + 20	7.68	1.49	19.46
σ_B_ [MPa]	Pine wood	20	-	77.7	8.93	11.50
		250	10 + 10	41.9	14.0	33.48
		250	10 + 20	37.5	9.19	24.53
	Pine wood, Si O_2_ impregnated	20	-	81.4	5.25	6.45
		250	10 + 10	47.8	14.0	29.30
		250	10 + 20	43.5	12.1	27.91
A_0_ [mm^2^]	Pine wood	20	-	100.12	0.43	
		250	10 + 10	95.23	1.9	1.99
		250	10 + 20	95.36	1.11	1.17
	Pine wood, Si O_2_ impregnated	20	-	102.04	0.83	0.82
		250	10 + 10	96.95	1.05	1.09
		250	10 + 20	96.27	1.2	1.25

**Table 3:** Parameters of shape and scale in the Weibull distribution.

Material	Temp. [°C]	Exp. Time [min]	Shape	Scale
Pine wood	20	-	0.431	322.02
	250	10 + 10	0.317	50.33
	250	10 + 20	0.387	49.984
Pine wood, SiO_2_ impregnated	20	-	0.495	382.66
	250	10 + 10	0.335	169.80
	250	10 + 20	0.332	119.05
